# Augmenting early stroke diagnosis with an eye-tracker

**DOI:** 10.3389/fneur.2025.1632939

**Published:** 2025-10-14

**Authors:** Mohamed Abul Hassan, Yan Zhuang, Mohammad Shifat-E-Rabbi, Chad M. Aldridge, Andrew M. Southerland, Gustavo K. Rohde

**Affiliations:** ^1^Department of Biomedical Engineering, University of California, Davis, Davis, CA, United States; ^2^Imaging and Data Science Lab, Department of Biomedical Engineering, University of Virginia, Charlottesville, VA, United States; ^3^Department of Electrical and Computer Engineering, University of Virginia, Charlottesville, VA, United States; ^4^Imaging and Data Science Lab, Department of Electrical and Computer Engineering, University of Virginia, Charlottesville, VA, United States; ^5^Department of Electrical and Computer Engineering, North South University, Dhaka, Bangladesh; ^6^Department of Neurology, University of Virginia, Charlottesville, VA, United States; ^7^Department of Public Health Sciences, University of Virginia, Charlottesville, VA, United States

**Keywords:** stroke, posterior circulation acute ischemic stroke, eye tracking–computer module, emergency medicine, Optokinetic Nystagmus (OKN) eye movements

## Abstract

Posterior circulation stroke (PCS) presents significant diagnostic challenges due to poorly localizing and non-specific symptoms, such as dizziness, nausea, and headache, which are often misattributed to benign conditions. This study introduces an innovative diagnostic tool that utilizes a machine learning algorithm-driven eye tracker to enhance early diagnosis of PCS. Our approach involves analyzing eye movements during three standard neurological eye examinations: the Dot Test, H Test, and Optokinetic Nystagmus (OKN) Test. The Discrete Radon Cumulative Distribution Transform (DRCDT) and nearest subspace (NS) classification methods were employed to distinguish between PCS patients and healthy controls by identifying specific eye movement patterns. Results demonstrate that the ensemble model combining the three tests achieved the highest sensitivity and accuracy, with a sensitivity of 96% and an accuracy of 88%, in diagnosing PCS. This study's findings underscore the potential of an eye-tracker-based diagnostic tool to support a more accurate and efficient diagnosis, particularly for non-neurology trained providers, which would improve patient outcomes with more timely and appropriate treatment. The proposed tool offers a practical solution to the limitations of current diagnostic methods, such as the need for calibration and reliance on highly trained specialists, and can be seamlessly integrated into clinical settings to support emergency medical services (EMS) and emergency department (ED) triage.

## 1 Introduction

Stroke is a significant contributor to mortality and long-term disability worldwide, with anterior circulation stroke (ACS) being the most prevalent type and posterior circulation stroke (PCS) being less common and often misdiagnosed (1–3). PCS can have non-specific and variable symptoms, such as dizziness, nausea, and vomiting, and headache, similar to other more commonly presenting conditions such as migraine, metabolic disturbances, infections, and peripheral vestibulopathies (4, 5). In contrast, the symptoms of ACS, including facial asymmetry, arm weakness, and speech disturbances, are more specific and easier to recognize as signs of stroke, particularly for non-neurology trained providers (6).

The HINTS (Head Impulse, Nystagmus, Test of Skew) examination is a bedside diagnostic approach to help differentiate PCS from an inner ear disorder in patients presenting with acute vestibular syndrome (7). However, the accuracy of the HINTS exam depends on a certain level of training and experience, which is often not the case for lay providers in the emergency setting (8–11). Thus, there is a need to develop augmented diagnostic tools not reliant on provider experience and training to help detect PCS in the emergency setting.

PCS may be differentiated from a peripheral disorder by abnormal patterns of eye movements, which are often subtle and difficult to distinguish from benign conditions. Commonly observed abnormalities in eye movements include nystagmus, fixed gaze deviation, and dysconjugacy, which neurologists and ophthalmologists are trained to examine at the bedside. For an automated diagnostic tool to accurately diagnose PCS, it would need to replicate the ability of a specialist to correctly discern these oculomotor abnormalities in real time.

Eye tracking has emerged as a promising tool for diagnosing neuro-ocular disorders. Eye tracking may be used to characterize various neurological conditions, including traumatic brain injury, Parkinson's disease, and multiple sclerosis (12–17). Pupillary light reflex (PLR) has also been proposed as a potential biomarker for neurological dysfunction, including in differentiating stroke patients from healthy individuals (18). However, PLR primarily reflects autonomic function and is not sensitive to vestibulo-ocular abnormalities, which are critical for identifying PCS. Our approach instead focuses on evaluating extra-ocular eye movements, such as saccades, smooth pursuit, and gaze-evoked nystagmus, which are more directly affected in PCS.

Previously, we demonstrated that a non-calibrated eye tracker can be used to assess eye movement symmetry and variability in PCS patients (19). The calibration procedure posed an obstacle to the translation of the eye tracker for clinical settings, as patients affected by stroke and other neurological conditions may have difficulty fixating on a target or following a moving object; i.e., the prerequisite for calibrating an eye tracker.

This study proposes a novel approach to augment the diagnosis of PCS using a commercial, off-the-shelf eye tracker. By analyzing eye movements during computer-adapted versions of three standard bedside oculomotor tests (i.e., the Dot-Test, H-Test, and OKN-Test), we aim to identify patterns to aid in the early and accurate diagnosis of PCS. Additionally, acquiring data from PCS patients is challenging due to the nature of their illness, which can affect patient compliance during the examination. To address these challenges, we will use a custom-built mobile rig equipped with compliance protocols for data acquisition. The study involves recruiting patients with PCS and healthy controls and recording their eye movements using an eye tracker during three oculomotor tests. These tests investigate gaze-evoked nystagmus, smooth pursuit, saccades, and the vestibular-ocular reflex. Given that PCS presents less commonly than anterior circulation stroke, our machine-learning algorithms must be data-efficient. The resulting eye-tracking data will be analyzed using advanced pattern recognition and machine learning algorithms to identify specific eye movement patterns characteristic of PCS.

To summarize, this work makes significant contributions:

- Proposing a novel diagnostic tool that utilizes a non-calibrated eye tracker combined with machine learning algorithms to accurately distinguish between posterior circulation stroke (PCS) patients and healthy controls. This tool addresses the limitations of current diagnostic methods, such as the need for calibration and the reliance on highly trained specialists.
- The systematic validation of the tool on a cohort of PCS patients and healthy controls demonstrated high sensitivity (96%) and accuracy (88%) to detect PCS, underscoring its potential for early and precise diagnosis.
- This diagnostic tool offers a practical solution that can be seamlessly integrated into clinical settings, supporting emergency medical services (EMS) and emergency department (ED) triage. By enhancing the accuracy and efficiency of PCS diagnosis, this tool holds promise to improve patient outcomes through timely and appropriate stroke treatment.

## 2 Materials and methods

Our approach utilizes the Discrete Radon Cumulative Distribution Transform (DRCDT) and nearest subspace (NS) classification model. The DRCDT is applied to gaze points from both left and right eye movements to maximize information on eye conjugacy while being invariant to deformation caused by non-calibrated eye tracking data. This method generates a convex set of gaze point distributions in the DRCDT space, making them linearly separable and thus suitable for classification using the NS model. These factors will contribute to the robustness and applicability of our work.

**Figure 1:**
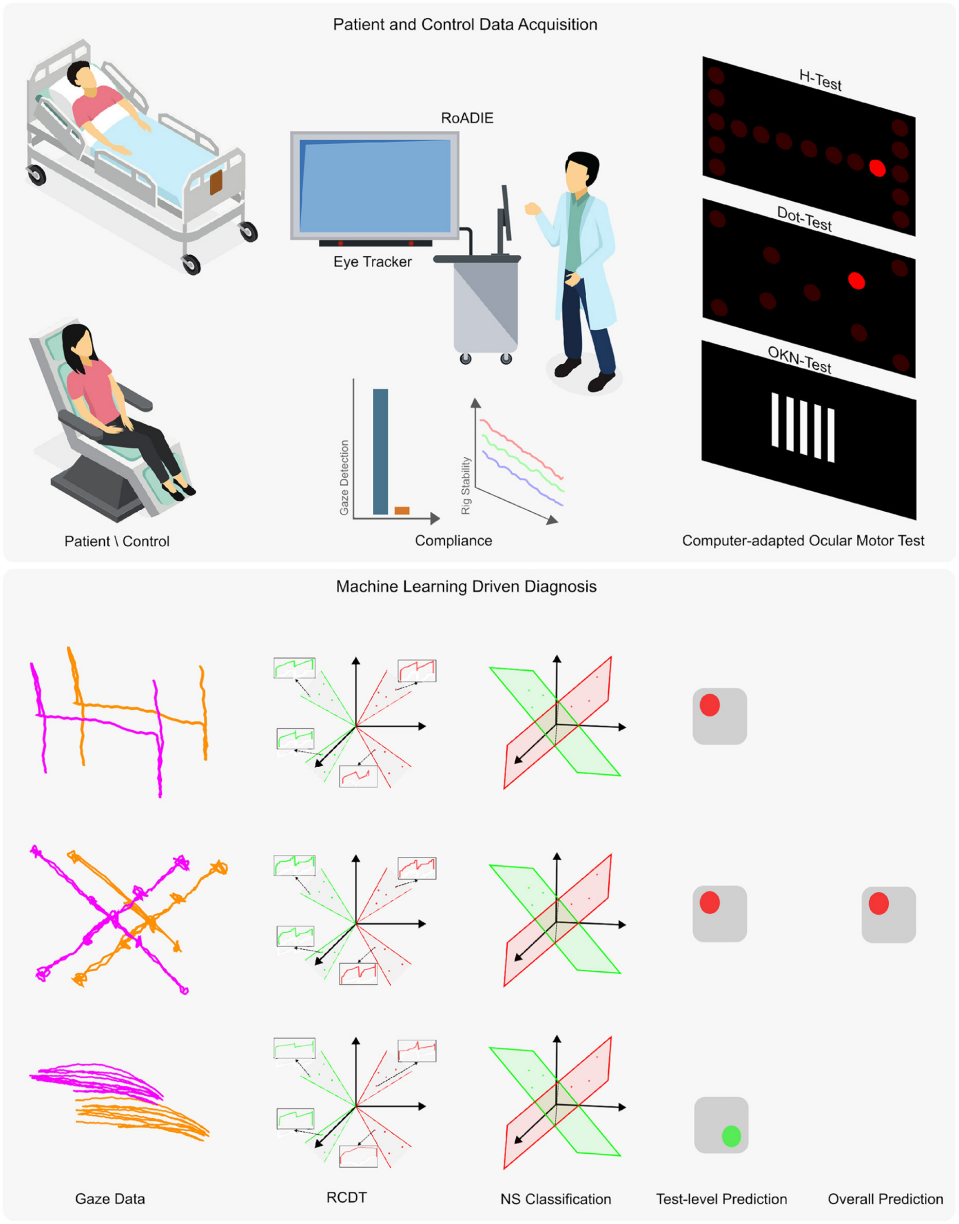
Overview of the proposed diagnostic tool using an eye tracker. The upper panel presents the data acquisition process, while the lower panel outlines the machine learning adaptation for automatic diagnosis from non-calibrated gaze data. Data were collected from patients and controls either reclined or seated. The Rolling Apparatus to Detect Impairment of the Eyes (RoADIE) is equipped with a screen-based eye tracker to monitor gaze during computer-adapted neurological eye examinations on an extended screen. Compliance with gaze detection and rig stability requirements is ensured (as shown in the upper panel, second column). The upper panel's third column illustrates the digitally adapted bedside ocular motor tests: H-test, Dot-test, and OKN-test. In the lower panel, the first column displays the gaze path captured by the eye tracker during the ocular motor tests. The second column shows the transformation of the gaze point data's “x” and “y” coordinates to the RCDT space. The third and fourth columns illustrate the three near-subspace classification models and their predictions for each ocular motor test. Finally, the fifth column depicts the ensemble model that combines the three individual models to provide a binary diagnosis.

The overall diagnostic tool comprised an ensemble model based on three separate DRCDT-NS classifiers for the individual tests: the Dot-Test, H-Test, and OKN-Test. Each of these neurological eye examinations interrogates different ocular motor abnormalities, and a separate diagnosis of PCS per test may result in the potential for false positives or negatives (see Figure 1). Therefore, the ensemble model allows for a more accurate and efficient diagnosis by combining the diagnostic power of the three neurological eye examinations. The results are compared to the final diagnosis of a vascular neurologist to assess the accuracy and efficiency of the proposed approach.

### 2.1 Human data acquisition

The experimental protocol was approved by the University of Virginia's Institutional Review Board. As such, the protocol complies with all national ethical research standards and in accordance with the Declaration of Helsinki. Written informed consent was obtained prior to subject enrollment and testing of hospitalized patients and healthy participants.

This study was conducted at the University of Virginia Medical Center. Participants were enrolled between May 2020 and May 2022 and were assessed at the bedside in the emergency department or inpatient unit settings.

Adults aged 18 years or older were eligible for inclusion. Patients were enrolled if they presented within 7 days of symptom onset with continuous dizziness lasting at least one hour. Dizziness types included lightheadedness, vertigo, disequilibrium, head motion intolerance, and spontaneous or gaze-evoked nystagmus. Exclusion criteria included prior history of vestibular or oculomotor disorders (e.g., vestibular migraine, benign paroxysmal positional vertigo), acute intoxication, recent head trauma, significant cognitive impairment, inability to provide informed consent, or resolution of symptoms before enrollment. Healthy participants were recruited based on the absence of vestibular, neurological, or oculomotor deficits.

Nineteen healthy participants were enrolled initially, with a mean age (range) of 40 (25–62) years, and 79% were female. The racial distribution of the healthy participants was 81% White, 14% Asian, and 5% American Indian or Alaska Native. One healthy control was excluded since the participant failed to follow instructions during the experiment and moved out of the required testing position, which prevented eye gaze capture, resulting in 18 healthy participants included in the analysis.

Comparatively, twenty-nine patients were screened, and of those, 27 enrolled. The patients consisted of 53% females with a mean age (range) of 61.8 (19–96) years. The patients had a racial distribution of 82% White, 12% Black, and 6% Other. Two patients were excluded from the analysis (acute ischemic stroke *n* = 1 and acute vestibular syndrome peripheral origin *n* = 1) due to malfunction of the hardware/software of RoADIE, resulting in 27 patients included in the analysis (see Table 1).

**Table 1:** Participant demographics, including the number of individuals screened and enrolled, mean age, gender, and racial distribution for healthy participants and patients.

**Group**	**Healthy participants**	**Patients**
Screened	19	29
Enrolled	18	27
Mean age (range)	40 (25–62)	61.8 (19–96)
Female (%)	79	53
White (%)	81	82
Asian (%)	14	0
American Indian or Alaska native (%)	5	0
Black (%)	0	12
Other (%)	0	6

Patient enrollment and data collection were completed within a median of 3 days, ranging from 0 to 37 days of symptom onset. Acute ischemic stroke accounted for 22 patients, with the rest having a diagnosis of Acute Vestibular Syndrome of suspected peripheral origin (*n* = 3), Multiple Sclerosis (*n* = 1), and Vestibular Neuritis (*n* = 1). Diplopia, or double vision, was the primary presenting symptom in six patients, with nystagmus or vertigo being the presenting characteristic in seven patients. Head impulse testing showed that seven patients had normal responses, while four had an impaired vestibular-ocular reflex. The head impulse test was not performed for six patients (see Table 2). None of the patients had acute hearing loss. Only four patients had impaired smooth pursuit.

**Table 2:** Distribution of patient diagnoses and presence of abnormal eye movement for enrolled participants used in the analysis.

**Diagnosis**	**Neurological symptoms**	**Population**	**Abnormal eye movement**
Acute ischemic stroke	Central	22	Yes
Hemorrhagic stroke	Central	1	Yes
Multiple sclerosis	Central	1	Yes
Acute vestibular syndrome	Peripheral	3	No
Healthy	None	18	No

Each participant underwent a single eye-tracking assessment, which required approximately 5 min to set up once the equipment was brought to the patient's location. The entire procedure lasted between 5 to 10 min. Trained study personnel performed the setup, while the RoADIE software conducted the test autonomously, delivering standardized auditory instructions and visual stimuli throughout the task.

## 3 Rolling apparatus to detect impairment of the eyes

The study employed a custom-built mobile rig designed to facilitate the integrated acquisition of diverse sensory data within a clinical environment (19, 20). The central component of this rig was the Tobii Pro Fusion Eye Tracker (21), which provided gaze estimates at a sampling frequency of 120 Hz. The setup also included a secondary screen for visual stimulation and a RealSense camera that captured RGB, infrared, depth, and motion data via an integrated accelerometer (see Figure 1).

To accommodate different patient conditions, the mobile rig was designed for easy transportation to a patient's bedside, allowing for data collection while the patient was either reclined or seated. For consistency in baseline measurements, participants from the control cohort were seated during data acquisition.

The data acquisition protocol was defined to ensure the reliability and precision of the collected data. It required the eye tracker to detect at least one eye and capture at least 90% of gaze data during neurological eye examination tests. Furthermore, the protocol leveraged the accelerometer to monitor the rig's stability, with a directive to halt data collection upon detecting any significant motion. In post-processing, the series of gaze points for each exam, ${\text{x}}_{k}\in {\mathbb{R}}^{2},k=1,\cdots \;,N$ is smoothed using a Savitzky-Golay filter with a window size of 30 points to remove artifacts including those caused by blinks and minor involuntary movement.

### 3.1 Computer-adapted neurological eye examinations

To quantitatively assess ocular motor function, three standard bedside ocular motor tests were adapted into computerized formats: the Dot Test, the H Test, and the Optokinetic Nystagmus (OKN) Test. These tests were designed to mimic the visual tracking and coordination tasks typically conducted by clinicians, with digital enhancements to measure and record eye movement accurately (19).

Dot Test: This test evaluates the quality of eye coordination in performing volitional saccades. Participants were instructed to shift their gaze from one dot to another as these appeared on the screen. This test replicates the clinical assessment of saccade accuracy, where a clinician observes if the eyes move sharply and stop precisely at the target. Key metrics for analysis include instances where the eyes undershoot or overshoot the target, or fail to initiate a saccade.

H Test: In this test, eye movements are evaluated as participants track a visual target moving in a pattern that resembles the letter “H.” This pattern requires the eyes to follow the target through the four cardinal ocular directions and quadrants, simulating the movement of following a clinician's finger during a physical examination. The test particularly assesses the smoothness of pursuit and the ability of the eyes to initiate and maintain motion in all directions. Abnormal findings are characterized by a lack of motion, delayed initiation, or the use of compensatory small saccades to maintain target tracking.

OKN Test: Measures the participant's ability to switch from a smooth pursuit to a saccade in order to fixate on the next visual target after the first target disappears. The visual stimulus is typically comprised of vertical bars with a high contrast to the background. The bars move at a quick and constant pace from right to left. This is done for each opposite direction. OKN typically remains preserved in individuals with occipital lobe infarcts, although impairment of a visual field may limit the amplitude of saccadic fixation. Asymmetry in the performance of the eyes or poor ability to generate saccades during the OKN test may suggest damage to the ocular, brainstem, or cerebellar nuclei or tracts.

### 3.2 Discrete radon cumulative distribution transform for eye movement classification

The Discrete Radon Cumulative Distribution Transform (DRCDT) (22) for pattern recognition and classification of eye movements using the nearest subspace (NS) classification approach. By analyzing the gaze points from both the left and right eyes, DRCDT maximizes the information on eye conjugacy while being invariant to the deformation caused by non-calibrated eye tracking data. This invariance allows for the generation of a convex set of gaze point distributions within the DRCDT space, which are then linearly separable and can be effectively classified using the NS model.

#### 3.2.1 Mathematical formulation of DRCDT

Let Ω_2_ refer to a set of coordinates in ℝ^2^ and *s*:Ω_2_ → ℝ be a mapping from Ω_2_ to a set of real numbers. The discrete point-set distribution *P*_*s*_ then can be defined as


(1) $$\begin{array}{l} P_{s}:=\frac{1}{\left|\Omega_{2}\right|}\underset{\text{x}\in \Omega_{2}}{\sum }\delta_{s\left(\text{x}\right)} \end{array}$$

where |·| denotes the cardinality of a set. The DCDT transform of a distribution *P*_*s*_ is defined as


(2) $$\begin{array}{l} F\left(P_{s}\right)=P{\left[s\left({\text{x}}_{1}\right),s\left({\text{x}}_{2}\right),\cdots \right]}^{T}={\left[\tilde{s}\left({\text{x}}_{1}\right),\tilde{s}\left({\text{x}}_{2}\right),\cdots \right]}^{T} \end{array}$$

where P is a permutation matrix such that $\tilde{s}\left({\text{x}}_{1}\right)\leq \tilde{s}\left({\text{x}}_{2}\right)\leq \cdots \;$. The Radon transform of a distribution *P*_*s*_ is defined as


(3) $$\begin{array}{l} P_{s\theta }:=\frac{1}{\left|\Omega_{2}\right|}\underset{\text{x}\in \Omega_{2}}{\sum }\delta_{s\left(\text{x}\cdot {\text{w}}_{\theta }\right)} \end{array}$$

where ${\text{w}}_{\theta }={\left(\cos\theta ,\sin\theta \right)}^{T}$ is a unit vector in the direction of θ. The DRCDT transform of a distribution *P*_*s*_ is defined as


(4) $$\begin{array}{l} {\hat{P}}_{s}\left(\theta \right)=F\left(P_{s\theta }\right):=P_{\theta }{\left[s\left({\text{x}}_{1}\cdot {\text{w}}_{\theta }\right),s\left({\text{x}}_{2}\cdot {\text{w}}_{\theta }\right),\cdots \right]}^{T}\\ \;={\left[\tilde{s}\left({\text{x}}_{1}\cdot {\text{w}}_{\theta }\right),\tilde{s}\left({\text{x}}_{2}\cdot {\text{w}}_{\theta }\right),\cdots \right]}^{T} \end{array}$$

where $P_{\theta }$ is a permutation matrix such that $\tilde{s}\left({\text{x}}_{1}\cdot {\text{w}}_{\theta }\right)\leq \tilde{s}\left({\text{x}}_{2}\cdot {\text{w}}_{\theta }\right)\leq \cdots \;$.

#### 3.2.2 Classification process

The classification of unknown test samples of gaze distributions uses the DRCDT space. During the training phase, the DRCDT transform of each training sample is calculated and used to approximate the subspace for each class: $\left\{P_{s_{1}}^{\left(1\right)},P_{s_{2}}^{\left(1\right)},\cdots \right\}$ (class 1), $\left\{P_{s_{1}}^{\left(2\right)},P_{s_{2}}^{\left(2\right)},\cdots \right\}$ (class 2) and the subspace can be approximated as,


(5) $$\begin{array}{l} V^{\left(k\right)}=\text{span}\left(\left\{{\hat{P}}_{s_{1}}^{\left(k\right)},{\hat{P}}_{s_{2}}^{\left(k\right)},\cdots \right\}\cup U_{T}\right) \end{array}$$

where *U*_*T*_ = {μ_1_(*n*, θ), μ_2_(*n*, θ)} with μ_1_(*n*, θ) = cosθ, μ_2_(*n*, θ) = sinθ is the spanning set corresponding to the deformation modeling.

In the testing phase, the class of an unknown test distribution *P*_*s*_ is obtained as

(6) $$\begin{array}{l} k^{*}=\arg\underset{k}{\min}d\left(P_{s},V^{k}\right) \end{array}$$

where *d*(·, ·) denotes the distance of a test sample from a trained subspace in the DRCDT transform space, and *k* refers to the class index.

### 3.3 Evaluation

We evaluated our model's ability to detect abnormal eye movement vs. health controls and patient sub-cohort that present with normal eye movement using the following metrics: Accuracy, Sensitivity, and Specificity. We did this for each Neurological eye examination test (Dot, H, and OKN) as well as the overall classification of abnormal eye movement vs. healthy control using all the information from these tests. This approach allowed us to determine how much value each test has in the aim of discriminating between abnormal eye movement vs. healthy controls. Additionally, we studied the classification performance with several time series neural network methods: 1D Visual Geometry Group (1D-VGG) (23), 1D Residual Network (1D-ResNet) (23, 24), Long Short Term Memory (1D-LSTM) (23).

## 4 Results

To evaluate binocular coordination, we computed Spearman correlation coefficients between the left and right eyes for each oculomotor test—DOT, H, and OKN—along both the X and Y axes. Table 3 summarizes the mean correlations and 95% confidence intervals for healthy controls and patients.

**Table 3:** Non-calibrated left–right eye Spearman correlations (mean and 95% CIs) for each test and axis.

**Test**	**Axis**	**Control (healthy)**	**Patient**
DOT	X	0.980 [0.976–0.985]	0.844 [0.602–0.920^*^]
	Y	0.948 [0.924–0.969]	0.922 [0.869–0.949]
H	X	0.979 [0.971–0.986]	0.903 [0.746–0.958^*^]
	Y	0.887 [0.869–0.907]	0.886 [0.834–0.919]
OKN	X	0.993 [0.987–0.996]	0.898 [0.785–0.958^*^]
	Y	0.706 [0.604–0.799]	0.581 [0.282–0.713]

^*^Statistical significance.

In the control group, Spearman correlations were consistently high across all tests and axes. For instance, the DOT test yielded near-perfect inter-eye correlation on the X-axis (0.980 [0.976–0.985]) and strong correlation on the Y-axis (0.948 [0.924–0.969]). Similar trends were observed for the H test (X: 0.979 [0.971–0.986]; Y: 0.887 [0.869–0.907]) and the OKN test (X: 0.993 [0.987–0.996]; Y: 0.706 [0.604–0.799]).

In contrast, patients exhibited reduced inter-eye correlation, particularly in the OKN and DOT tests. Notably, OKN Y-axis coordination was markedly lower in patients (0.581 [0.282–0.713]) compared to controls (0.706 [0.604–0.799]). DOT X-axis performance also showed reduced coordination (0.844 [0.602–0.920]) relative to controls (0.980 [0.976–0.985]). While the H test showed somewhat preserved coordination (e.g., Y: 0.886 [0.834–0.919]), the confidence intervals indicate greater variability in the patient group.

These findings demonstrate that patients with acute vestibular or neurological disorders exhibit measurable impairments in inter-eye coordination across several oculomotor paradigms, with the most pronounced effects observed in reflexive tracking tasks like OKN. This highlights the clinical utility of automated eye-tracking metrics in distinguishing pathological oculomotor behavior.

### 4.1 Fully automated PCS diagnosis tool using eye-tracker

The validation of the method for early-stage stroke diagnosis encompassed two sets of results: (1) Cohort 1: Stroke patients with abnormal eye movement (*n* = 24) vs. healthy controls (*n* = 18), (2) cohort 2: Stroke patients with abnormal eye movement (*n* = 24) vs. healthy controls (*n* = 18) and stroke patients with no abnormal eye movement (*n* = 3) (see Table 4). The model for cohort 1 is trained with healthy controls and patients with central neurological symptoms (i.e., exhibiting abnormal eye movements). The analysis from the 10,000 bootstrapped participant-level classification performance of cohort 1 shows that the ensemble model using data from the eye tracker of all three neuro-ophthalmological tests reported the highest sensitivity and accuracy in diagnosing posterior circulation stroke, with a sensitivity of 0.96 and accuracy of 0.88 (see Table 4). The analysis from cohort 2, trained and validated with patients with abnormal eye movement as the positive label and healthy controls along with stroke patients with peripheral neurological symptoms (no abnormal eye movement) reported no change in sensitivity for the ensemble model. However, reported a minor decrease in accuracy and specificity 0.87 and 0.76 respectively.

**Table 4:** Mean with 95% confidence Interval (CI) of classification performance for each test and ensemble model from 10,000 bootstrapped participant-level sensitivity, specificity, and accuracy.

**Model**	**Accuracy**	**Sensitivity**	**Specificity**
**Patients with abnormal eye movement vs. Healthy controls**			
Dot	0.83 [0.71–0.93]	0.83 [0.67–0.96]	0.83 [0.65–1.0]
H	0.67 [0.52–0.81]	0.5 [0.29–0.7]	0.89 [0.72–1.0]
OKN	0.71 [0.57–0.86]	0.54 [0.33–0.74]	0.94 [0.81–1.0]
**Ensemble model**	**0.88 [0.79–0.98]**	**0.96 [0.86–1.0]**	**0.78 [0.57–0.95]**
**Patients with abnormal eye movement vs Healthy controls and**			
**Patients with no abnormal eye movement**			
Dot	0.82 [0.71–0.93]	0.83 [0.67–0.96]	0.81 [0.63–0.96]
H	0.69 [0.56–0.82]	0.5 [0.3–0.7]	0.9 [0.76–1.0]
OKN	0.73 [0.6–0.84]	0.54 [0.33–0.74]	0.95 [0.85–1.0]
**Ensemble model**	**0.87 [0.76–0.96]**	**0.96 [0.86–1.0]**	**0.76 [0.57–0.94]**

Bold rows indicate the ensemble model, which represents the overall best-performing approach by combining results across individual tests (Dot, H, and OKN) to provide improved classification performance.

**Figure 2:**
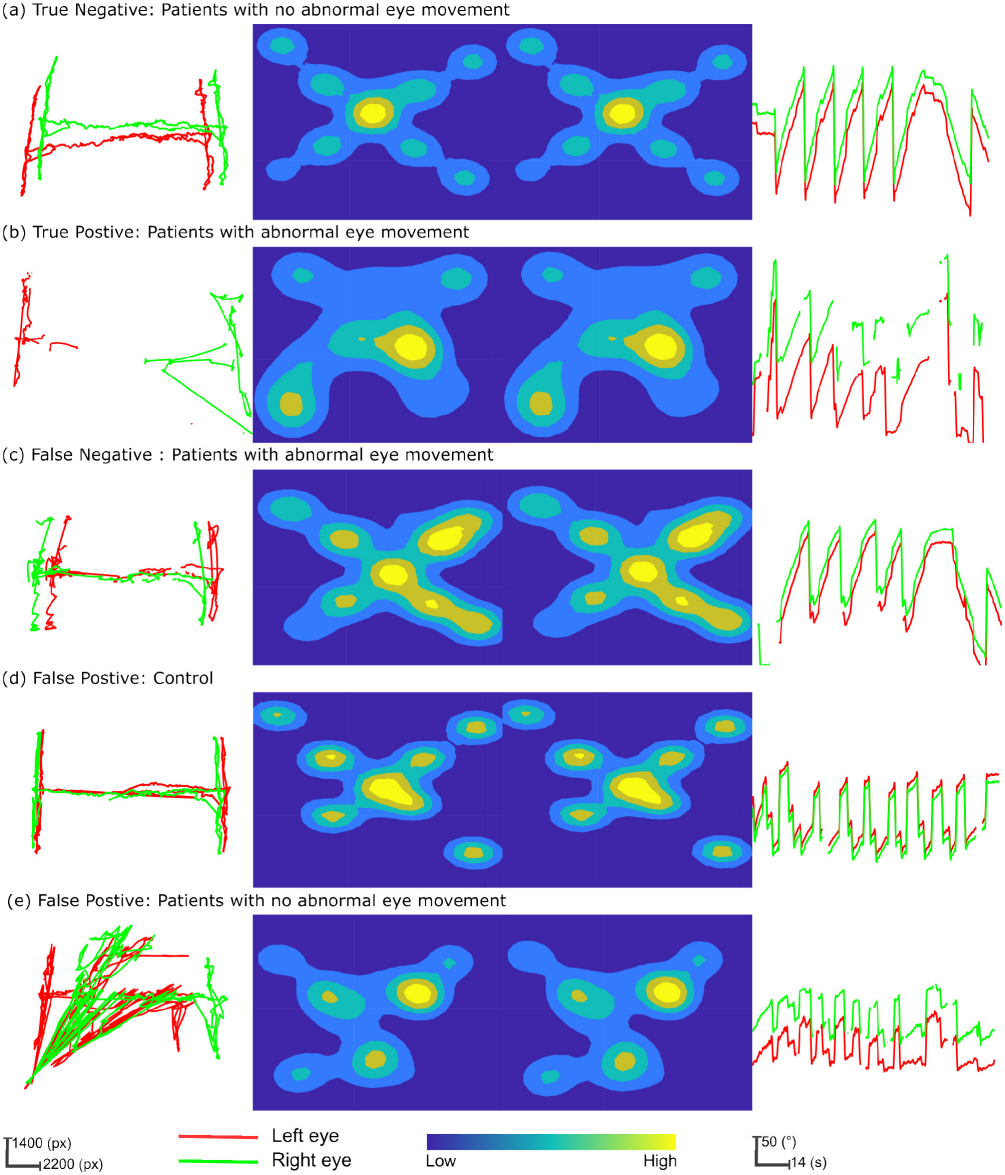
Multifaceted eye-tracking data visualization for classification prediction analysis. The columns of the figure represent each neurological eye examination and the rows depict instances of gaze patterns, integrating True Positives, True Negatives, False Positives, and False Negatives in the context of patient and control characteristics. The first column depicts the path plot from the “H-test.” Here gaze data is transformed into screen coordinates measured in pixels (px). The central columns feature heatmaps for the “Dot-test,” with the left and right eyes' fixation densities portrayed through a color spectrum, where warmer colors denote higher fixation densities and cooler colors indicate less frequent fixations. The final column provides relative angle plots from the “OKN test,” sequentially plotted over time, to reveal the rhythmic eye movements characteristic of nystagmus. **(a)** True negative: patients with no abnormal eye movement. **(b)** True positive: patients with abnormal eye movement. **(c)** False negative: patients with abnormal eye movement. **(d)** False positive: control. **(e)** False positive: patients with no abnormal eye movement.

The path plot and heatmap for the three neurological exams Figures 2a, b illustrate the contrast in gaze data from patients with abnormal eye movement and normal eye. The true positive classification of predicting abnormal eye movement as positive in a patient Figure 2b, and the true negative classification of predicting a patient with normal eye movement as negative. The path plot of the true positive instance with a high degree of variation in the horizontal axis with two distinctly different shapes and orientations of clusters (much pronounced in the H-test) illustrates the cause of pathology during the eye examinations. The Figure 2c illustrates a false negative classification instance of a patient with abnormal eye movement as negative. The path plot, and heat map for the three neurological exams showed normal conjugate eye movement as referenced to Figure 2a and overall normal eye movement distribution of this study. This led to the point that the pathology of this patient was not detected by the eye examinations performed during this study. The false positive prediction originating primarily from the Dot-test Figures 2d, e and H-test Figure 2e. The gaze data from these exams shows eye conjugacy where both eyes move together. However, the gaze data from the control and patient did not follow the overall normal distribution as shown from the instance Figure 2a. Therefore the classification model classified the gaze pattern out of the normal distribution as abnormal eye movement. This probes the question of compliance during the eye examination.

The overall results irrespective of the participant cohort indicate that the ensemble model comprising models specific to each eye examination reported the highest classification performance. Considering the individual tests, the Dot Test reported the highest sensitivity and accuracy after the ensemble model, with a sensitivity of 0.83 and an accuracy of 0.83. The individual tests reported a higher specificity compared to the ensemble model. Clinicians often prioritize higher sensitivity over specificity, as avoiding a false negative diagnosis is more important than a false positive in the clinical scenario. A false negative diagnosis may lead to delayed treatment, increased morbidity, and mortality, while a false positive diagnosis can be corrected with further testing or follow-up. These results further underscore that the combination of our proposed pattern recognition approach with the eye tracker has the potential to provide a more accurate and efficient diagnosis.

### 4.2 Ablation study and comparison of model performance

In our ablation study (see Table 5), we examined cohort 1, consisting of stroke patients with abnormal eye movements, and compared them to healthy individuals. Our focus was on evaluating two versions of the proposed DRCDT-NS model: one that incorporates deformation modeling and one that does not. Our findings indicate that omitting the deformation vector from the model's learning parameters led to a decrease in the model's accuracy. Specifically, the version of the DRCDT-NS model that omitted deformation modeling showed reduced sensitivity and specificity across tests, with a notably lower specificity of 0.55 in the combined analysis of the three tests (i.e., ensemble).

**Table 5:** Ablation study and comparison of model performance for patients with abnormal eye movement vs. healthy controls.

**Model**	**Test**	**Accuracy**	**Sensitivity**	**Specificity**
1D VGG	Dot	0.711	0.852	0.500
	H	0.738	0.833	0.611
	OKN	0.690	0.792	0.556
	Ensemble	0.643	0.958	0.222
1D ResNet	Dot	0.578	0.741	0.333
	H	0.714	0.792	0.611
	OKN	0.595	0.958	0.111
	Ensemble	0.643	0.958	0.222
LSTM	Dot	0.689	0.926	0.333
	H	0.643	0.833	0.389
	OKN	0.667	0.833	0.444
	Ensemble	0.595	1.000	0.056
DRCDT-NS	Dot	0.711	0.704	0.722
	H	0.744	0.721	0.778
	OKN	0.786	0.792	0.778
	Ensemble	0.733	0.851	0.555
DRCDT-NS + deformation modeling	Dot	0.822	0.814	0.833
	H	0.622	0.444	0.889
	OKN	0.666	0.481	0.944
	Ensemble	0.866	0.925	0.778

This drop in performance was attributed to a higher occurrence of false positives, suggesting that the model struggles to distinguish between abnormal and normal eye movements when deformation is not considered. This outcome aligns with the results from the comparative analysis involving another deep learning model, which similarly showed low specificity in its ensemble predictions. However, it's worth mentioning that models like the 1D-VGG and 1D-ResNet demonstrated promising results for individual assessments, such as the H-test and the Dot-test.

The aggregate prediction is crucial for the accurate detection of disease, as it leverages the unique ocular motor functions assessed by each test, which may be affected by the condition in question. A model that minimizes false positives is key to developing a diagnostic tool with significant practical utility. In this context, the DRCDT-NS model, when it includes deformation modeling, proves to be particularly effective, offering high classification accuracy.

## 5 Discussion

Clinically, PCS is three times more likely to be misdiagnosed than anterior circulation stroke (2). In our study, we demonstrated that a non-calibrated eye tracker, augmented with ML can effectively diagnose PCS by analyzing eye movements during neurological examinations. This method was applied to a population of stroke patients exhibiting abnormal eye movements compared with healthy controls and stroke patients without abnormal eye movements. These results are particularly significant as the requirement for calibration has traditionally been a major barrier to the adoption of eye-tracking technology in clinical diagnosis. Previous studies exploring the application of eye tracking in various neurological disorders have often had to exclude patients unable to complete the calibration process, either fully or partially (13, 17, 25–30). Our approach addresses this limitation, broadening the potential for eye tracking in clinical settings.

The proposed approach overcomes the calibration by measuring the conjugacy of the eye movement instead of measuring the point of gaze coordinates concerning the screen coordinates. The lack of calibration introduces deformation to the measurement translation, scaling, rotation, and shearing. These deformations pose challenges during discriminative analysis. The DRCDT accounts for these deformations by modeling them as a deformation vector derived from the probability distribution of gaze point gradients. Excluding the deformation vector as a learning parameter degrades the robustness of the classification model. Augmenting early detection of PCS using an non-calibrated eye tracker enables paramedics and other emergency medical providers to more accurately perform early stroke screening and improve rapid stroke triage and treatment.

Vascular neurologists in our study diagnosed patients with a range of signs including impaired convergence, oculomotor palsies, skew deviation, abnormal patterns of nystagmus, impaired vestibulo-ocular reflex, saccadic pursuits, and acute hearing loss. Given the complexity of these variable presentations, with 48% of patients exhibiting multiple symptoms with abnormal nystagmus being the most prevalent, an ensemble model that combines multiple tests is essential for diagnostic robustness. The neurological eye examination adopted in this study assesses both singular deficits and syndromes with multiple deficits. Relying solely on the results of a single test could be inadequate due to the need to fully characterize and localize a PCS syndrome. This is underscored by the low sensitivity of the H-test and OKN-tests alone, as detailed in Table 4. Furthermore, the classification prediction analysis illustrated in Figure 2 shows that models based on a single test may yield false negatives when the manifested symptom does not align with the specific test used.

The computer-adapted tests provide a controlled, repeatable, and precise method to assess complex eye movements and coordination, which are critical in diagnosing and monitoring the nervous system. The integration of these tests into the study's methodology allows for detailed analysis of oculomotor function in a way that is not feasible with traditional bedside tests alone. The examinations included tests for visual fields and eye movements, such as the ability to maintain a steady gaze and detecting abnormal patterns of nystagmus. However, our methodology did not encompass specific tests for diplopia, or double vision, that often arises from strokes affecting the cranial nerves or brainstem. We incorporated the Head Orientation (HO) test, which required participants to alternately tilt their heads to the left and right. This test is known to exacerbate vertical misalignment due to an ocular tilt reaction. Nonetheless, the HO test was ultimately excluded from our methodology because it showed poor gaze correlation coefficients among both patients and healthy participants and exhibited higher variance compared to the other three tests used (19). Moreover, our observations indicated that screen-based eye-tracking technology was inadequate for this test, as it failed to detect gaze accurately when the head's orientation was rotated along the y-axis.

Rigorous quality control measures during data acquisition required multiple test attempts, especially within the patient cohort, highlighting the challenges of maintaining consistency and reliability in a clinical setting. Despite these difficulties, strict adherence to the protocol was crucial for securing high-quality data. This underscores the efficacy of the integrated technological approach employed in our study.

**Figure 3:**
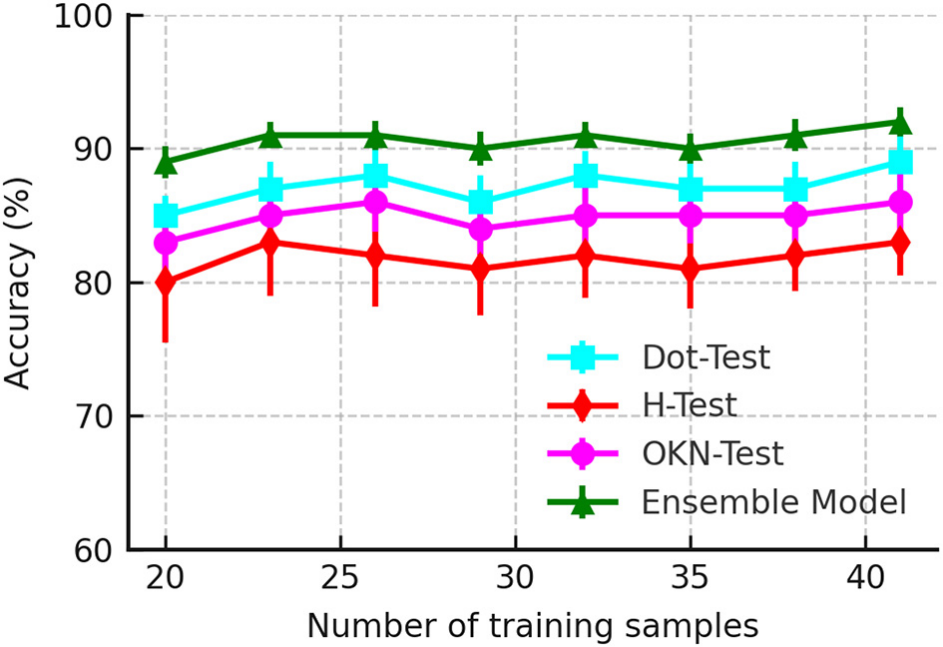
Accuracy as a function of a number of training samples used to evaluate the classifier performance.

Data efficiency is critical in solving the clinical problem as clinical data for PCS is challenging to acquire due to its limited incidence. Furthermore, acquiring a clinical dataset is more expensive than other domains' datasets. We evaluated the data efficiency of the DRCDT-based classification method by performing a k-fold cross-validation by varying the training sample. The k-folds are randomly drawn from the original dataset. The experiment for each k-fold was repeated five times, and the results were averaged. Figure 3 highlights the computational efficiency of the classification model as it maintains classification accuracy over the varying number of training samples. This is feasible since the classifier utilizes a transport-based generative model to define the classification problem and uses the mathematical properties of the DRCDT to render the problem more accessible in the transform domain. This approach allows one to solve nonlinear classification problems using linear classifiers.

We observed that 81% of participants in our study presented with dizziness, a non-specific symptom widely manifested in other conditions. Non-specific symptoms like dizziness, nausea, and blurred vision increase the likelihood of diagnostic errors in PCS (31). Diagnostic error is exacerbated by the fast-paced, high-pressure environment of the emergency department, leading clinicians to rely on heuristics and rapid decision-making prone to cognitive biases (31–33). Diagnostic error rates in PCS can reach up to 52%, with non-specific symptoms like dizziness and visual disturbances being common reasons for misdiagnosis (34, 35). Furthermore, despite the introduction of more advanced imaging techniques, diagnostic errors still contribute to significant clinical and socioeconomic consequences, including higher rates of disability, increased mortality, and longer hospital stays (31, 35). This study is proof of concept that a non-calibrated eye tracker using machine learning techniques could augment detection of PCS by non-neurology providers and emergency personnel, improve patient care and outcomes through early recognition, triage, and more effective stroke treatment.

## Data Availability

The raw data supporting the conclusions of this article will be made available by the authors, without undue reservation.

## References

[1] Go S. Posterior circulation ischemic stroke. Mo Med. (2015) 112:192. 26168589 PMC6170115

[2] Types of Stroke. American Stroke Association. (2021). Available online at: https://www.stroke.org/en/about-stroke/types-of-stroke (Accessed May 21, 2023).

[3] Olivato S Nizzoli S Cavazzuti M Casoni F Nichelli PF Zini A. e-NIHSS: an expanded National Institutes of Health Stroke Scale weighted for anterior and posterior circulation strokes. J Stroke Cerebrov Dis. (2016) 25:2953–7. 10.1016/j.jstrokecerebrovasdis.2016.08.011 27693107

[4] Kmetonyova S Schwabova JP Sramkova T Dankova M Olserova A Petrzalka M. Posterior circulation stroke diagnosis in unselected group of acutely dizzy patients. Clin Neurol Neurosurg. (2023) 224:107541. 10.1016/j.clineuro.2022.107541 36493551

[5] Zhuang Y Hassan M Aldridge C Yin X McMurry T Southerland A. “A pilot study on camera-based neurological deficit detection,” in *ACM/IEEE International Conference on Connected Health: Applications, Systems and Engineering Technologies (CHASE '20), December 16-18, 2020, Arlington, VA, USA*. ACM/IEEE (2020). p. 16–17.

[6] Salerno A Strambo D Nannoni S Dunet V Michel P. Patterns of ischemic posterior circulation strokes: a clinical, anatomical, and radiological review. Int J Stroke. (2022) 17:714–22. 10.1177/17474930211046758 34581223 PMC9358301

[7] Kattah JC Talkad AV Wang DZ Hsieh YH Newman-Toker DE. HINTS to diagnose stroke in the acute vestibular syndrome: three-step bedside oculomotor examination more sensitive than early MRI diffusion-weighted imaging. Stroke. (2009) 40:3504–10. 10.1161/STROKEAHA.109.551234 19762709 PMC4593511

[8] Pula JH Yuen CA. Eyes and stroke: the visual aspects of cerebrovascular disease. Stroke Vasc Neurol. (2017) 2:210–20. 10.1136/svn-2017-000079 29507782 PMC5829892

[9] Gurley KL Edlow JA. Avoiding misdiagnosis in patients with posterior circulation ischemia: a narrative review. Acad Emerg Med. (2019) 26:1273–84. 10.1111/acem.13830 31295763

[10] Nakatsuka M Molloy EE. The HINTS examination and STANDING algorithm in acute vestibular syndrome: a systematic review and meta-analysis involving frontline point-of-care emergency physicians. PLoS ONE. (2022) 17:e0266252. 10.1371/journal.pone.0266252 35511910 PMC9070939

[11] Krishnan K Bassilious K Eriksen E Bath PM Sprigg N Brækken SK. Posterior circulation stroke diagnosis using HINTS in patients presenting with acute vestibular syndrome: a systematic review. Eur Stroke J. (2019) 4:233–9. 10.1177/2396987319843701 31984230 PMC6960692

[12] Terao Y Fukuda H Hikosaka O. What do eye movements tell us about patients with neurological disorders? An introduction to saccade recording in the clinical setting. Proc Japan Acad Series B. (2017) 93:772–801. 10.2183/pjab.93.049 29225306 PMC5790757

[13] Kumar D Dutta A Das A Lahiri U. Smarteye: developing a novel eye tracking system for quantitative assessment of oculomotor abnormalities. IEEE Trans Neural Syst Rehabilit. Eng. (2016) 24:1051–9. 10.1109/TNSRE.2016.2518222 26780816

[14] Oyama A Takeda S Ito Y Nakajima T Takami Y Takeya Y. Novel method for rapid assessment of cognitive impairment using high-performance eye-tracking technology. Sci Rep. (2019) 9:1–9. 10.1038/s41598-019-49275-x 31506486 PMC6736938

[15] Asfaw DS Jones PR Edwards LA Smith ND Crabb DP. Using eye movements to detect visual field loss: a pragmatic assessment using simulated scotoma. Sci Rep. (2020) 10:1–13. 10.1038/s41598-020-66196-2 32555198 PMC7299979

[16] Rosengren W Nyström M Hammar B Stridh M. A robust method for calibration of eye tracking data recorded during nystagmus. Behav Res Methods. (2019) 52:36–50. 10.3758/s13428-019-01199-0 30825158 PMC7005097

[17] Grillini A Ombelet D Soans RS Cornelissen FW. “Towards using the spatio-temporal properties of eye movements to classify visual field defects,” in *Proceedings of the 2018 ACM Symposium on Eye Tracking Research* & *Applications*. (2018). p. 1–5. 10.1145/3204493.3204590

[18] Scala I Miccoli M Pafundi PC Rizzo PA Vitali F Bellavia S. Automated pupillometry is able to discriminate patients with acute stroke from healthy subjects: an observational, cross-sectional study. Brain Sci. (2024) 14:616. 10.3390/brainsci14060616 38928617 PMC11202086

[19] Hassan MA Aldridge CM Zhuang Y Yin X McMurry T Rohde GK. Approach to quantify eye movements to augment stroke diagnosis with a non-calibrated eye-tracker. IEEE Trans Biomed Eng. (2022) 70:1750–1757. 10.1109/TBME.2022.3227015 37015585

[20] Hassan MA Yin X Zhuang Y Aldridge CM McMurry T Southerland AM. A digital camera-based eye movement assessment method for NeuroEye examination. IEEE J Biomed Health Inf . (2023) 28:655–665. 10.36227/techrxiv.21767825.v1

[21] AB T. Tobii Pro Fusion. Reston: Tobii Pro Inc. (2019).

[22] Zhuang Y Li S Yin X Rubaiyat AHM Rohde GK. Local sliced-wasserstein feature sets for illumination-invariant face recognition. arXiv preprint arXiv:220210642. (2022). 40061222 10.1016/j.patcog.2025.111381 PMC11884647

[23] Iwana BK Uchida S. An empirical survey of data augmentation for time series classification with neural networks. PLoS ONE. (2021) 16:e0254841. 10.1371/journal.pone.0254841 34264999 PMC8282049

[24] Fawaz HI Forestier G Weber J Idoumghar L Muller PA. Data augmentation using synthetic data for time series classification with deep residual networks. arXiv preprint arXiv:180802455. (2018).

[25] Rosengren W Nystöm M Hammar B Stridh M. “Suitability of calibration polynomials for eye-tracking data with simulated fixation inaccuracies,” in *Proceedings of the 2018 ACM Symposium on Eye Tracking Research* & *Applications*. (2018). p. 1–5. 10.1145/3204493.3204586

[26] Zeng Z Siebert FW Venjakob AC Roetting M. Calibration-free gaze interfaces based on linear smooth pursuit. J Eye Mov Res. (2020) 13:3. 10.16910/jemr.13.1.3 33828782 PMC7881880

[27] Oh AJ Chen T Shariati MA Jehangir N Hwang TN Liao YJ. A simple saccadic reading test to assess ocular motor function in cerebellar ataxia. PLoS ONE. (2018) 13:e0203924. 10.1371/journal.pone.0203924 30403759 PMC6221255

[28] Bijvank JN Van Rijn L Balk L Tan H Uitdehaag B Petzold A. Diagnosing and quantifying a common deficit in multiple sclerosis: internuclear ophthalmoplegia. Neurology. (2019) 92:e2299–308. 10.1212/WNL.0000000000007499 31004067 PMC6598816

[29] Bijvank JAN Petzold A Coric D Tan HS Uitdehaag BM Balk LJ. Quantification of visual fixation in multiple sclerosis. Invest Ophthalmol Visual Sci. (2019) 60:1372–83. 10.1167/iovs.18-26096 30938772

[30] Nij Bijvank J Petzold A Balk L Tan H Uitdehaag B Theodorou M. A standardized protocol for quantification of saccadic eye movements: DEMoNS. PLoS ONE. (2018) 13:e0200695. 10.1371/journal.pone.0200695 30011322 PMC6047815

[31] Hoyer C Szabo K. Pitfalls in the diagnosis of posterior circulation stroke in the emergency setting. Front Neurol. (2021) 12:682827. 10.3389/fneur.2021.682827 34335448 PMC8317999

[32] Newman-Toker DE Schaffer AC Yu-Moe CW Nassery N Saber Tehrani AS Clemens GD. Serious misdiagnosis-related harms in malpractice claims: the “Big Three”–vascular events, infections, and cancers. Diagnosis. (2019) 6:227–40. 10.1515/dx-2019-0019 31535832

[33] Graber ML Franklin N Gordon R. Diagnostic error in internal medicine. Arch Intern Med. (2005) 165:1493–9. 10.1001/archinte.165.13.1493 16009864

[34] Saber Tehrani AS Lee H Mathews SC Shore A Makary MA Pronovost PJ. 25-Year summary of US malpractice claims for diagnostic errors 1986–2010: an analysis from the National Practitioner Data Bank. BMJ Quality Safety. (2013) 22:672–80. 10.1136/bmjqs-2012-001550 23610443

[35] Madsen TE Khoury J Cadena R Adeoye OM Alwell KA Moomaw CJ. Potentially missed diagnosis of ischemic stroke in the emergency department in the Greater Cincinnati/Northern Kentucky Stroke Study. Acad Emer Med. (2016) 23:1128–35. 10.1111/acem.13029 27313141 PMC5358009

